# Examining the daily relationship between guilt, shame, and substance use among veterans with psychiatric disorders

**DOI:** 10.1016/j.dadr.2023.100174

**Published:** 2023-06-15

**Authors:** Pallavi Aurora, Stefanie T. LoSavio, Nathan A. Kimbrel, Jean C. Beckham, Patrick S. Calhoun, Kirsten H. Dillon

**Affiliations:** a Durham VA Health Care System, Durham NC, USA; b VA Mid-Atlantic Mental Illness Research, Education and Clinical Center, Durham, NC, USA; c Department of Psychiatry and Behavioral Sciences, The University of Texas Health Science Center at San Antonio, San Antonio, TX, USA; d Department of Psychiatry and Behavioral Sciences, Duke University School of Medicine, Durham, NC, USA; e VA Health Services Research and Development Center of Innovation to Accelerate Discovery and Practice Transformation, Durham, NC, USA

**Keywords:** Substance use, Guilt, Shame, Veterans

## Abstract

**Background:**

Shame and guilt are key emotions known to amplify trauma-related symptoms in veterans. Maintenance of symptoms is facilitated by avoidance behaviors, such as substance use. However, limited research has examined the associations between shame, guilt, and substance use in daily life.

**Methods:**

The current study sought to examine the cross-lagged association between shame, guilt, and substance use. Forty veterans completed 28 days of experience sampling reporting on their current emotional experiences and use of substances.

**Results:**

Results suggest a reciprocal relationship among shame and guilt and substance use, such that shame and guilt separately predicted subsequent substance use, and substance use predicted subsequent shame and guilt.

**Conclusions:**

These results highlight the dynamic relationship among shame, guilt, and substance use and suggest the potential value of conceptualizing these clinical targets as mutually reinforcing to inform integrative intervention strategies that can interrupt the in-the-moment cascade of negative consequences.

## Highlights

• Shame and guilt are associated with the maintenance of psychiatric disorders.
• Limited research has examined shame, guilt, and substance use in daily life.
• Findings suggest a reciprocal relationship between shame/guilt and substance use.
• Understanding the bi-directional relationship may help inform future treatment.

Problematic substance use is a growing concern among military veterans, with recent prevalence rates reaching up to 43% for heavy drinking, 5% for prescription medication misuse, and 11% for illicit drug use (Hoggatt et al., 2017). Substance use is associated with several deleterious outcomes and is also commonly associated with psychiatric disorders (e.g., PTSD, depression) as well as maladaptive regulatory behaviors (e.g., non-suicidal self-injury [NSSI]; Blakey et al., 2020; Teeters et al., 2017). The use of substances has been commonly understood as a behavior utilized to dampen unwanted physiological and emotional experiences (Aurora and Coifman, 2020; Blakey et al., 2020; Weiss et al., 2015). Furthermore, by providing short-term relief, it reinforces the use of substances in the future (Clark, 1999; Elhers & Clark, 2000). For example, findings suggest that the use of substances to manage heightened negative emotions subsequently increases the risk of substance use in the future, often resulting in long term consequences (Held et al., 2015; Kubany and Watson, 2003; Wilson et al., 2006). Although there is a breadth of cross-sectional and longitudinal research examining the relationships between substance use and emotions, there are limited data on these associations as they occur in the daily life of veterans with psychiatric diagnoses. Given the high prevalence of substance use in veteran populations, it is imperative to understand the emotional processes driving the maintenance of substance use to better inform treatment.

Two key emotional experiences commonly theorized to be associated with substance use are shame and guilt (Dearing et al., 2005; Kroll & Egan, 2004; Luoma et al., 2018). Shame is characterized by a tendency to feel bad about oneself (e.g., “I am not good enough”), whereas guilt is characterized by a tendency to feel bad about a specific behavior (e.g., “I feel bad about drinking”). Several theories including the negative reinforcement model (Baker et al., 2004) and tension reduction models (Cooper et al., 1995) suggest that elevations in negative emotions, such as shame and guilt, precede the use of substances, as a means to down-regulate or cope with the negative emotional experience. However, research testing the relationships between shame and guilt and substance use has been mixed. Some cross-sectional studies have found a positive association between shame and substance use (Stuewig et al., 2015; Tangney & Dearing, 2002) and guilt and substance use (Kubany and Watson, 2003; Locke et al., 2015), others have found negative associations between shame and substance use (Grynberg et al., 2017), and guilt and substance use (Hequembourg and Dearing, 2013), and finally some studies have found no association among shame and guilt and substance use (Dodge & Clarke, 2018). Additionally, a recent meta-analysis by Luoma and colleagues (2019) found no association between shame and substance use. Despite these mixed findings, there has been consistent findings that both shame and guilt are associated with drug and alcohol related problems (Luoma et al., 2019) such as increased drinking-related behavior, earlier onset of drinking behavior, and increased rates of relapse (Dearing et al., 2005; Locke et al., 2015; Luoma et al., 2017, 2018). In addition to these consequences, difficulties regulating negative emotions, such as shame and guilt, have also been associated with other maladaptive regulatory behaviors (e.g., NSSI) and psychiatric diagnoses (e.g., PTSD, Major Depression; Cunningham, 2020; Bannister et al., 2019; Sheehy et al., 2019). Indeed, high comorbidity rates among substance use and other maladaptive behaviors and psychiatric disorders is prevalent (Wolfe and Maisto, 2000; Killeen et al., 2015; Buckner et al., 2008). Thus, given the lack of clarity about the relationship between shame, guilt, and substance use and the known consequences with other diagnostic symptoms and behaviors, it is imperative to further examine these relationships.

Although the theoretical understanding of the associations among shame, guilt, and substance use is robust, these factors have rarely been examined together. Additionally, research has primarily focused on testing these associations cross-sectionally, with few studies examining these associations longitudinally. One longitudinal study by Batchelder and colleagues (2022) examined the relationship between shame and guilt with stimulant use over 15 months. Their findings suggested that high initial levels of shame predicted increased difficulties or slower decreases in stimulant use over time, and initial levels of guilt and alcohol use were positively related. Additionally, there have only been a few studies that have tested these associations using ecological momentary assessment (EMA). In EMA, constructs are assessed just a few hours apart, providing an opportunity to understand how processes unfold in daily life. In a study by Mohr and colleagues (2008), participants were asked to report their emotional experiences of shame and alcohol use once a day for 21 days. Findings from their study suggested that shame predicted drinking at home and away from home in a sample of college students. However, these findings should be interpreted with caution as Mohr and colleagues (2008) only measured emotion and alcohol use once per day, compared to EMA studies which measure emotion and behavior several times during one day, allowing for a greater understanding of how behavior is affected during the day. Extending on the Mohr and colleagues’ findings, Luoma and colleagues (2018) were interested in testing both within- and between-person shame predicting solitary versus social drinking using experience sampling methods, across 21 days, with a sample of community members. Findings from their study found that both within- and between-person shame predicted solitary drinking. Although these findings begin to shed light on the longer-term and daily relationships between emotions and substances, to our knowledge, there are no studies examining the reciprocal relationship between shame and guilt and substance use using EMA in a clinical sample. The use of EMA provides a better understanding of the temporal associations between guilt, shame, and substance use, and thus we can gain insight into ways to improve treatment interventions, which typically target daily or more frequent behaviors. The aim of the present study was to examine the reciprocal relationships between shame and guilt and substance use in a clinical sample of veterans with psychiatric diagnoses using EMA to better capture the associations in daily life. Using a multilevel cross-lagged approach we were able to test our primary hypothesis that shame and guilt would predict subsequent substance use and substance use would predict subsequent shame and guilt.

## 1 Method

### 1.1 Participants and procedure

This study was a secondary analysis of an EMA study on veterans with Non-suicidal Self Injury (NSSI) disorder (Dillon et al., 2021a, 2021b). The present study sample was a subset of a larger project that was designed to study the impact of NSSI on functional outcomes in veterans (see Dillon et al., 2021a for full description of larger project). To be eligible to participate in the EMA portion of the study (the present study), participants had to be veterans, 18 years or older, and meet the criteria for NSSI disorder. Veterans with other mental health diagnoses were included in the study with exclusion criteria including lifetime bipolar or psychotic spectrum disorder or imminent risk for suicide or homicide. Forty participants enrolled in this EMA study. Of these participants 72.5% were male, with a mean age of 46.7 (*SD* = 12.75). Regarding race, 55% identified as Black and 45% identified as White. The lifetime prevalence for PTSD and major depressive disorder were both 95% and the current prevalence for PTSD was 90% (*n* = 36) and major depressive disorder was 82.50% (*n* = 33) in the current sample. All study procedures were approved by the Durham VA Institutional Review Board (IRB) and Research and Development Committee.

### 1.2 Measures

#### 1.2.1 Diagnostic measures

NSSI disorder was assessed using the Clinician Administered Non-Suicidal Self-Injury Disorder Index (CANDI; Gratz et al., 2015), which has demonstrated good interrater reliability (*k* = 0.83) and adequate internal consistency (α = 0.71; Gratz et al., 2015). The CANDI is a semi-structured interview that assesses each of the DSM-5 criteria for NSSI disorder. Participants who met criteria for the diagnosis were included in the current study.

Diagnostic Interviewing was also conducted to assess other potential comorbid psychiatric diagnoses using the Structured Clinical Interview for DSM-5 (SCID-5; First et al., 2016), which has demonstrated good interrater reliability (*k* = 0.84), sensitivity, and specificity (Osório et al., 2019). Interviews were conducted by Masters-level clinicians, who completed extensive training and participated in ongoing weekly supervision.

### 1.3 Ecological momentary assessment procedures

Participants used an Android smartphone for 28 days of EMA data collection. At an initial training session, participants set a 14-hour wake period and a 10-hour sleep period. Alarmed prompts were active during the wake period and inactive during the sleep period. During the day, participants received three random alarms (approximately four hours apart), which prompted them to answer questions about their affective states, NSSI urges and behaviors, current activity and setting, and substance use in the past four hours. In addition to these prompted entries, participants were instructed to initiate an experience sampling entry if they engaged in NSSI or felt the urge to do so. For a detailed description of the experience sampling methodology please see Dillon et al., 2021a.

Items from the Positive and Negative Affect Schedule-Expanded Form (PANAS-X; Watson and Clark, 1999) were used to assess affect. Among other affective states, participants rated the extent to which they felt *guilty* and *ashamed* during the past four hours on a scale of 0 (*Not at all*) to 4 (*Extremely*). A between-person shame and guilt score was calculated (Shame: *M* = 0.85, *SD* = 0.98; Guilt: *M* = 0.88, *SD* = 1.04) by deriving the mean across participants, and a within-person shame and guilt score was calculated by subtracting the person mean. Additionally, a between-person (*M* = 1.13, *SD* = 0.75) and within-person negative affect score was derived by summing the following affect words across the diary: *upset, afraid, sad*, and *angry*. They were also asked “*In the past 4 h, have you engaged in other potentially self-destructive behaviors?”* They could select from the following responses: “*No, I have not; Used alcohol; Used drug/pills; Misused prescription medications; Spent impulsively; Binge eating; Purging; Engaged in unsafe sex; Other(describe).”* If participants endorsed using alcohol, drugs/pills, or misusing prescription medications, they were counted as having engaged in substance use. A dichotomous variable was created to measure the presence or absence of engaging in substance use. If participants endorsed at least one of the substances, the variable was coded as the presence of substance use. Substance use was endorsed a total of 239 times across the experience sampling for all participants with a mean count endorsement of *M* = 8.43 (*SD* = 13.40) times per person.

Participants were compensated based on their level of compliance with EMA procedures. If they completed more than 75% of the prompted entries, they were paid $250; completing 50–74% received $170; 25–49% received $100; and 0–25% received $50. High rates of compliance were achieved: Mean compliance was 81.6%, and the mean number of prompted experience sampling entries was 68.57 (*SD* = 16.54). When including self-initiated entries, participants completed a mean of 86.35 (*SD* = 15.90) total entries. Analyses of compliance did not find any demographic (i.e., age, gender, race) or clinical variables (i.e., PTSD or MDD status) to be associated with the number of diaries completed (*p*-values > 0.09).

### 1.4 Data analytic plan

Due to the hierarchical nature of the data and our interest in examining the current and lagged relationships between affect and behavior, a multilevel modeling analysis with path analysis was employed to examine the direction of the association of shame and guilt with substance use over time using SAS 9.4 and Mplus 7. The use of path analysis allows for the examination of both the autoregressive effects of shame and guilt predicting substance use and whether substance use predicts subsequent shame and guilt. By controlling for autoregressive effects, we can more reliably estimate change over time while controlling for the previous completed diary signal. There are seven key assumptions of the cross-lagged models that were considered in our analyses: 1) synchronicity, 2) stationarity, 3) comparing cross-lagged coefficients, 4) measurement error, 5) timeframe effect, 6) omitted variables and 7) stability (see Kearney, 2017). To account for missing data, we utilized statistical software by using all data that was available to estimate the model using full information maximum likelihood. Therefore, data that is missing is excluded from the analyses following the listwise deletion method. Lastly, to consider stability and the impact of inter-individual differences, other relevant variables (e.g., sex, PTSD) were considered, however due to lack of effects on the relationships of interests these analyses were not reported. Due to the nature of our variables, substance use as a categorical variable, and guilt/shame as a count variable, we used the specifications in Mplus to determine the best fitting distribution for our model. Based on model fit statistics, the best fitting model was identified as substance use as a categorical variable with no additional distribution specifications and guilt/shame as a count variable with a Poisson distribution.

**Fig. 1:**
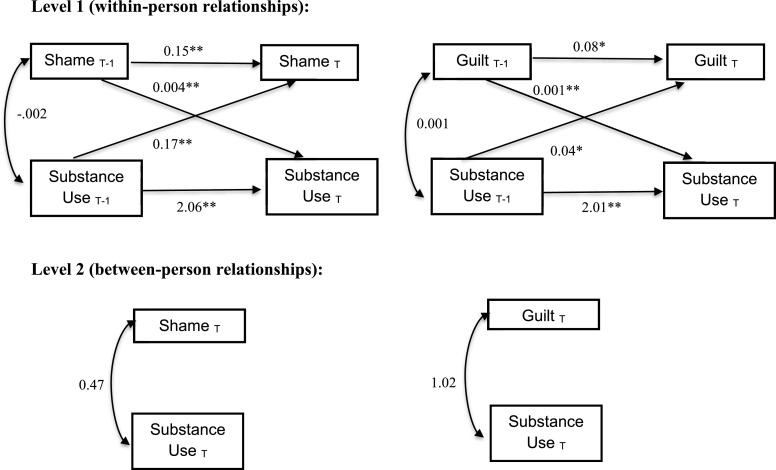
Cross-lagged path models of shame and guilt and substance use.

Two analyses were conducted testing the cross-lagged path model (Fig. 1) among between-person and within-person shame predicting substance use and between-person and within-person guilt predicting substance use (See Table 1 for bivariate correlations). To account for the influence of time on behavior and affect, only diaries completed within 6 h of the next completed experience sampling entry were included in the analysis. This resulted in inclusion of an average of 53.40 (*SD* = 15.80) diaries per person.

**Table 1:** Summary of bivariate correlations for key variables.

Variables	1	2	3	4	5
1) Guilt	1	–	–	–	–
2) Shame	.879*	1	–	–	–
3) Rate of Pills use	.178	.242	1	–	–
4) Rate of Alcohol use	.230	.115	.211	1	–
5) Rate of Misuse Meds use	.083	.007	.053	.066	1

⁎ *p*<.001.

## 2 Results

### 2.1 Main analysis

First, we examined the associations between shame and substance use. Between-person results indicated that between-person shame was not a significant predictor of substance use. Within-person results indicated that the lagged effect of shame on subsequent substance use was significant (*B* = 0.004, *p* < .001) and the lagged effect of substance use on subsequent shame was also significant (*B* = 0.17, *p* = 0.004), such that as there were increases in shame within a person, from signal to signal, there was an increased likelihood of substance use in the subsequent signal and as substance use increased from signal to signal, there was an increased likelihood of shame in the subsequent signal. Additionally, lagged substance use was significant in predicting subsequent substance use (*B* = 2.06, *p* <0.001) and lagged shame was a significant predictor of subsequent shame (*B* = 0.15, *p* < .001).

Next, we were interested in examining the associations between guilt and substance use. Between-person results indicated that between-person guilt was not a significant predictor of substance use. Within-person results indicated that the lagged effect of guilt on subsequent substance use was significant (*B* = 0.001, *p* <0.001) and the lagged effect of substance use on subsequent guilt was also significant (*B* = 0.04, *p* = 0.029) such that as there were increases in guilt within a person, from signal to signal there was an increased likelihood of substance use in the subsequent signal and as substance use increased from signal to signal, there was an increased likelihood of guilt in the subsequent signal. Additionally, lagged substance use was a significant predictor of subsequent substance use (*B* = 2.01, *p* <0.001) and lagged guilt was a significant predictor of subsequent guilt (*B* = 0.08, *p* = .03).

### 2.2 Exploratory analysis

Given that the results were similar for both shame and guilt, we wanted to explore whether combining shame and guilt together may help better describe the relationship between shame and guilt and substance use. Between-person results for shame + guilt combined indicated that shame + guilt was not a significant predictor of substance use (*B* = 0.15, *p* = .51). Within-person results indicated that shame + guilt was a significant predictor of substance use (*B* = 0.36, *p* < .001), and substance use was also a significant predictor of shame + guilt (*B* = 1.03, *p* < .001).

Additionally, we were interested in examining whether the effects of the shame and guilt analysis would hold when controlling for negative affect (sum of upset, afraid, sad, and angry). Between-person results indicated that when controlling for negative affect, guilt was not a significant predictor of substance use (*B* = 0.90, *p* = .12). Within-person results indicated that guilt was no longer a significant predictor of substance use (*B* = 0.002, *p* = .98) and substance use was also no longer a significant predictor of guilt (*B* = 0.04, *p* = .45). Additionally, negative affect was a significant predictor of guilt (*B* = 0.43, *p* < .001), and substance use (*B* = 0.74, *p* < .001). Between-person results for shame indicated that shame was not a significant predictor of substance use (*B* = 0.48, *p* = .28) when controlling for negative affect. Within-person results indicated that shame was no longer a significant predictor of substance use (*B* = 0.04, *p* = .67), and substance use was no longer a significant predictor of shame (*B* = 0.13, *p* = .24). Additionally, negative affect was a significant predictor of shame (*B* = 0.54, *p* < .001) and substance use (*B* = 0.33, *p* = .001).

Lastly, we were interested in testing whether our primarily analysis results would hold when controlling for the other affect variable in the model. First, we ran the model with shame and substance use while controlling for guilt. Between-person results indicated that shame was not a significant predictor of substance use (*B* = 0.14, *p* = .83) when controlling for guilt. Within-person results indicated that shame was still a significant predictor of substance use (*B* = 0.09, *p* = .001), and substance use was also still a significant predictor of shame (*B* = 0.008, *p* < .001) when controlling for guilt. Next, we ran the model with guilt and substance use while controlling for shame. Between-person results indicated that guilt was not a significant predictor of substance use (*B* = 3.12, *p* = .38). Within-person results indicated that guilt was still a significant predictor of substance use (*B* = 0.13, *p* = .02), however, substance use was not a significant predictor of guilt (*B* = 0.04, *p* = .78) when controlling for shame.

## 3 Discussion

We used EMA to examine the cross-lagged relationships between guilt and shame and substance use among veterans with NSSI disorder, the vast majority of whom (95%) also met diagnostic criteria for PTSD and major depressive disorder. Results indicated that both shame and guilt predicted subsequent substance use, and that substance use also predicted both subsequent guilt and shame. These results were also mostly consistent when we controlled for the other emotion variables in the analyses. These findings provide evidence of a dynamic, reciprocal relationship between these self-conscious emotions and substance use on a daily level. These relationships provide support for a functional and mutually maintaining relationship between these emotions and substance use unfolding in daily life. The reciprocal relationships observed between these variables may partially explain how these mood states and maladaptive coping strategies might reinforce one another over time. Indeed, our findings that shame and guilt predicted subsequent substance use is perhaps not surprising and is consistent with a tension-reduction formulation (Cooper et al., 1995) as well as the self-medication hypothesis (Khantzian, 1985; 1997), which suggests that substance use occurs to manage painful negative emotions. It is also consistent with prior literature indicating that shame predicts later drinking (e.g., Batchelder et al., 2022; Giguere et al., 2014; Luoma et al., 2018; Mohr et al., 2008; Randles and Tracy, 2013). However, this study expands our current understanding of this relationship as it unfolds on a daily level among a clinical sample of veterans.

It is also interesting that substance use predicted subsequent experiences of shame and guilt. This suggests that although substances may be used to dampen experiences of shame and guilt, paradoxically, it may actually facilitate more of these painful emotions. This is consistent with a cyclical characterization of the relationship, especially noted between shame and substance use, whereby shame serves as both a contributor and effect of substance use and addiction (Batchelder et al., 2022; Giguere et al., 2014; Luoma et al., 2018; Wiechelt, 2007). It may be the case, as considered in prior research, that individuals using substances to cope subsequently feel guilt and shame over their choices related to substance use (Fjaer, 2015; Giguere et al., 2014; Luoma et al., 2018). Or it may be the case that excess use of substances enhances one's experience of pre-existing negative thoughts and mood states (Kubany and Watson, 2003). Indeed, it is important to highlight that when we controlled for negative affect in our model, the effects of guilt and shame were no longer significant. This might suggest that the emotion behavior relationship might be driven more broadly by heightened negative emotional experiences in general, rather than by specific emotions. Although this finding is not surprising, it is important for us to consider the benefits of looking at specific emotions and their relationship to behavior. Indeed, there is a growing body of literature suggesting that although negative emotions broadly are associated with increased engagement in substance use, identification of specific emotions (i.e., emotion differentiation) may serve as a protective factor against continued engagement in these behaviors (Seah et al., 2020), such that the more an individual is able to identify and label different emotions the less likely they are to use maladaptive regulatory behaviors to down regulate negative affective experiences.

Recognition of a dynamic, reciprocal relationship between these variables in the daily life of veterans with NSSI, PTSD, and depression may inform potential clinical interventions. The current findings suggest that it is important to educate patients on the interactions of substance use and emotions such as guilt and shame. It may surprise patients to learn that substance use does not typically have the intended effect of reducing negative emotions in the long term, even though it might provide initial short-term relief and thus results in continued negative affective experiences. Clinicians could engage clients in discussing if they have found this to be the case in their experience and how they think they should manage negative emotions in the future. Indeed, our findings suggest that guilt and shame might be operating similarly and thus a broader understanding of the emotion behavior relationship could help veterans to recognize that guilt and shame may be a trigger for substance use and offer strategies to reduce their reliance on substance use as a coping mechanism. This discussion could lead to a focus on in-the-moment strategies to address guilt and shame and thus influencing the within-person fluctuations in emotions and behavior that influence one another. For example, as part of a cognitive approach, clinicians might teach veterans to become aware of their thoughts when they start feeling guilt and shame and to complete a worksheet to examine those thoughts. Indeed, in a meta-analysis review by Weiss et al. (2022), it was suggested that understanding one's relationship and response to emotions might be more beneficial in reducing reliance on behaviors such as substance use compared to engaging in specific strategies. Thus, an emphasis of psychoeducation about one's emotions and responses to emotions might help improve current interventions and treatment outcomes. The dynamic relationship observed here also points to the potential benefit of interventions focused on the treatment of comorbid conditions simultaneously (e.g., problematic substance use and PTSD; Back et al., 2019; Flanagan et al., 2016; Najavits, 2005). The current results suggest that such integrative approaches might be more appropriate given the interplay between negative mood states and substance use.

The current findings should be interpreted in light of the study's strengths and weaknesses. A major strength of the current investigation was the use of EMA techniques that allow examination of “life as it is lived” (Bolger et al., 2003). This methodology permits study of the dynamic processes at play on a daily or even more frequent level, offering better understanding of how variables relate. Additionally, the current study is one of the first of its kind to examine the associations between shame and guilt with substance use within one sample, and thus provides us with information on how these emotions might be unfolding together. It should be noted that the entire sample had NSSI disorder. This is a strength of the current study because it examined the relationship between these important variables in a clinical sample of veterans. It is unknown, however, the degree to which these findings would generalize to other clinical populations including civilians or veterans with PTSD but not NSSI. It seems likely, based on theory and past research (e.g., Batchelder et al., 2022; Luoma et al., 2018), that similar findings would be observed in other populations, but, by definition, the NSSI sample would be characterized by a higher tendency to self-soothe painful emotions with harmful coping strategies. Future studies should examine these research questions in more samples to determine the generalizability of the results. Nonetheless, the current findings are important for understanding veterans with NSSI disorder and PTSD and point to potential clinical considerations with this population. The current study also used a brief assessment battery to minimize burden for EMA data collection. However, future research would benefit from more in-depth examination of the emotion states and type and degree of substance use. Specifically, the measurement of emotion may benefit from more than one-item to increase reliability of the emotion being assessed (Leer, Lee, Smith & Luoma, 2022). The current measure of shame and guilt was based on single item measures for each emotion, which were highly correlated and thus our ability to suggest that these constructs are uniquely distinct is limited. Additionally, it is important to note that alcohol use might have been underreported in the diary due to the specific question asking participants if they engaged in “self-destructive behaviors.” Specifically, if participants did not believe that their alcohol use was self-destructive, they might not have endorsed the behavior. Thus, we must interpret the endorsement of alcohol use throughout the diary with caution and consider a potential for underreporting. Finally, future research should also capitalize on EMA to examine the effectiveness of integrative treatment interventions targeting guilt, shame, and substance use in the moment.

## 4 Conclusions

The relationships between guilt and shame and substance use were observed to be dynamic and reciprocal among veterans with NSSI, PTSD, and depression, with guilt and shame predicting subsequent substance use, and vice versa, on a daily level. These results highlight the potential value of conceptualizing these clinical targets as mutually reinforcing and using integrative intervention strategies that can interrupt the in-the-moment cascade of negative consequences.

## Declaration of Competing Interest

The authors declare that they have no known competing financial interests or personal relationships that could have appeared to influence the work reported in this paper.
